# Mental health problems and resilience in adolescents during the COVID-19 pandemic in a post-armed conflict area in Colombia

**DOI:** 10.1038/s41598-023-35789-y

**Published:** 2023-06-16

**Authors:** Carlos Gómez-Restrepo, María José Sarmiento-Suárez, Magda Alba-Saavedra, María Gabriela Calvo-Valderrama, Carlos Javier Rincón-Rodríguez, Lina María González-Ballesteros, Victoria Bird, Stefan Priebe, Francois van Loggerenberg

**Affiliations:** 1 Faculty of Medicine, Pontificia Universidad Javeriana, Bogotá, Colombia; 2 Unit for Social and Community Psychiatry, Queen Mary University of London, London, UK

**Keywords:** Psychology, Health care, Medical research

## Abstract

The impact of COVID-19 pandemic on mental health of adolescents are emerging and require particular attention in settings where challenges like armed conflict, poverty and internal displacement have previously affected their mental wellbeing. This study aimed to determine the prevalence of anxiety symptoms, depressive symptomatology, probable post-traumatic stress disorder and resilience in school-attending adolescents in a post-conflict area of Tolima, Colombia during the COVID-19. A cross-sectional study was carried out with 657 adolescents from 12 to 18 years old, recruited by convenience sampling in 8 public schools in the south of Tolima, Colombia, who completed a self-administered questionnaire. Mental health information was obtained through screening scales for anxiety symptoms (GAD-7), depressive symptomatology (PHQ-8), probable post-traumatic stress disorder (PCL-5) and resilience (CD-RISC-25). The prevalence observed for moderate to severe anxiety symptoms was 18.9% (95% CI 16.0–22.1) and for moderate to severe depressive symptomatology was 30.0% (95% CI 26.5–33.7). A prevalence of probable post-traumatic stress disorder (PTSD) of 22.3% (95% CI 18.1–27.2) was found. The CD-RISC-25 results for resilience had a median score of 54 [IQR 30]. These results suggest that approximately two-thirds of school-attending adolescents in this post-conflict area experienced at least one mental health problem such as anxiety symptoms, depressive symptomatology or probable PTSD during the COVID-19 pandemic. Future studies are of interest to establish the causal relationship between these findings and the impact of the pandemic. These findings highlight the challenge that schools have after pandemic to address the mental health of their students in order to promoting adequate coping strategies and implement prompt multidisciplinary interventions to reduce the burden of mental health problems in adolescents.

## Introduction

Since the COVID-19 outbreak was declared a pandemic by the World Health Organization in March of 2020, it has caused a worldwide emergency that has affected almost all human activity^1,2^. It is known that sudden public health emergencies, as well as armed conflicts, force individuals into situations where their coping abilities can be overwhelmed, thus considerably impacting on their mental health^3,4^. Lockdowns and physical distancing as risk-mitigating measures have brought unprecedented psychological, social and financial challenges to the global population^1^.

These challenges have modified social contexts and exacerbated existing problems, which is particularly worrisome for the mental health of people living in countries where big issues, such as poverty, armed conflict and internal displacement, were already being faced^5^. The undeniable adversities that the COVID-19 pandemic has caused, and their impact, require careful assessment. Younger populations like adolescents living in these contexts are facing this problem at a difficult age filled with key physical, social and psychological changes^6^. Measures to reduce the spread of the virus—such as lockdowns, physical distancing and school closures—have not only altered family and peer relationships and distanced young people from social spaces like classrooms and games, but also disrupted daily routines and their learning process and education. All of these changes might translate into mental health repercussions which can be particularly profound in vulnerable adolescents who are victims of armed conflict and forced displacement^7^.

Prior to the onset of the pandemic, it was estimated that globally, among those between 10 and 19 years of age, one in seven had experienced a mental health disorder, accounting for 13% of the global burden of disease in this age group^8,9^. It was widely acknowledged that the mental health needs of this population were often overlooked, especially in those exposed to poverty, abuse or violence^9,10^. International crises, like the conflict affecting Syria, have brought to light the consequences that violence and sudden disasters have on the mental health of young people: early exposure to adverse experiences precedes negative short-term and long-term effects on physical and mental health^11–14^. The most frequent associations between mental distress and armed conflict include post-traumatic stress disorder (PTSD), anxiety, depression and sleep disorders^10,14,15^.

In Colombia, as a country with a long history of armed conflict, the situation is worryingly. By 2020, It was estimated that there were approximately 20 million people aged 25 years or younger in the Colombian territory, which represents almost 40% of the population^16^. According to the 2015 National Mental Health Survey (NMHS-2015), the lifetime prevalence of any mental health disorder for those 12–17 years old was 7.2%. Additionally, according to the Self-Reporting Questionnaire (SRQ) the prevalence of depressive or anxiety symptoms in adolescents was found to be 12.2%^17^.

The armed conflict that has affected Colombia for over 50 years has imposed a heavy toll on the mental health of this population. According to the NMHS-2015, 29.3% of the population aged 12–17 years of age has experienced at least one traumatic event in their lives. In this age group, 18.3% of adolescents report having suffered forced displacement due to the armed conflict and insecurity. The percentage of individuals with a positive SQR score for anxiety and depressive symptoms among displaced adolescents was almost twice the percentage of non-displaced adolescents with positive results^17,18^.

The impact of the armed conflict varied across the geography of the territory, with one of the most affected regions being the department of Tolima in the central region of the country. Its location is crucial in the fight for territorial control related to drug trafficking due to the corridor it provides between the Colombian Pacific region and the centre of the country^19^. After peace agreements were signed between the Colombian government and the Revolutionary Armed Forces of Colombia-People's Army (known by its Spanish acronym of FARC-EP) in 2016, the most violence-affected territories were designated as part of the Development Program with Territorial Approach (PDET by its Spanish acronym) in order to prioritise the municipalities that suffered the most and to accelerate their transformation and access to services^20^. Within Tolima, the most affected rural municipalities were Chaparral, Rioblanco, Planadas and Ataco; these are territories where the guerrillas originated from and where multiple violent acts were committed. Armed attacks on the population, recruitment of minors, kidnappings, extortion, landmines, forced displacement, homicides and sexual violence were some of the forms of violence exercised by illegal armed groups in this part of the country. According to the Unit for the Victims, by August 2022 in this four municipalities, 36,602 victimizing events had been registered in Rioblanco, 33,104 in Chaparral, 31,927 in Planadas and 27,842 in Ataco^21^. The human rights violations committed during the conflict fragmented the integrity of the population, its cultural practices, its economic activities and its dignity^20^.

The armed conflict in Colombia has left 9,361,995 victims who have registered in the Single Registry of Victims^21^. Of these 1,051,668 have been adolescents between 12 and 17 years old. Unfortunately, after the peace agreement, the violence has not completely ceased in the territories. Although the numbers are lower than in the years prior this agreement, between 2016 and 2020, 992,360 new victims were registered. Other illegal armed groups and the dissidents of the extinct FARC-EP guerrilla have continued the struggle for territory, attacking the population. In 2021, of the total number of the victims in the department of Tolima, 14.38% were adolescents between 12 and 17 years old^21^.

This study attempts to describe mental health problems of adolescents in this post-conflict area during the COVID-19 pandemic considering, that its impacts on education, mental wellbeing, family life, income, among others, may have amplified the vulnerability of this population. The aim of the study was to determine the prevalence of anxiety symptoms, depressive symptomatology, probable post-traumatic stress disorder and resilience in adolescents attending public schools in a post-conflict area of Tolima, Colombia during the COVID-19.

## Materials and methods

### Study design and participants

This observational cross-sectional study was conducted on a sample of adolescent students from 8 public schools located in the municipalities of Chaparral, Rioblanco, Planadas and Ataco, who are part of the Development Program with Territorial Approach (PDET by its Spanish acronym) or the Southern Tolima in Colombia. In these municipalities, at the time of the study, there were 10 public schools in the urban area, attended by 4631 students in basic secondary and middle education. We include the schools that accept the invitation to participate in the study. The study was carried out between October 2021 and March 2022, once the Colombian government allowed the return of students to schools.

Assuming the prevalence of 21% anxiety and depressive symptomatology in adolescents reported by the Colombian National Mental Health Survey (NMHS-2015) for the Colombian central region, with a precision of 3% and 95% confidence level, the calculated sample size required was 615 participants. We calculated a sample size for PDET municipalities as one region.

A total of 657 participants aged between 12 and 18 were recruitment using a convenience sampling. There were no exclusion criteria. All participants completed the self-administered questionnaire that was delivered to them in person by the research team.

### Procedure

Principals of public schools in municipalities of Chaparral, Rioblanco, Planadas and Ataco were summoned through the Secretary of Education of Tolima and invited to participate in the study. The project coordinator informed them about the objectives of the study and the importance of their participation. Eight schools showed interest and were included. In a coordinated effort with the schools, an invitation to participate was extended to parents or legal guardians of students between 12 and 18 years of age. They were informed by the researches about the study’s objectives, the methods of data collection, the potential benefits and drawbacks of participation, privacy and confidentiality principles and the researcher’s contact information. Those who accepted the participation of their children or represented signed the informed consent. Students who had the informed consent of their parents or legal representatives were given the same information as their parents. The research team ensured that the students were aware of the voluntary nature of their participation and their right to withdraw from the study at any time, and to request that their personal data be removed from the analysis without any academic repercussions. Informed assent was obtained from those students who agreed to participate in the study.

Data collection was carried out by the research team face-to-face at each school at different times considering the challenges imposed by the pandemic, the school calendar and the personal safety of the research team in the municipalities. Participants were given a self-administered questionnaire with questions about their sociodemographic data and the following screening scales validated for the Colombian population: the family APGAR^22,23^, the Self-Report Questionnaire (SRQ)^24^, the Patient Health Questionnaire (PHQ-8)^25^, the Generalized Anxiety Disorder Scale (GAD-7)^26^, the Post-Traumatic Stress Disorder Checklist for DSM-5 (PCL-5)^27^ and the Connor and Davidson Resilience Scale (CD-RISC-25)^28^. The data collected was recorded by the research team in the REDCap (Research Electronic Data Capture) platform of the Pontificia Universidad Javeriana, keeping custody, confidentiality and anonymity of the data.

### Measurements

A group of professionally trained research assistants helped participants complete the sociodemographic questionnaire data and screening scales for family functionality, mental health symptoms, depressive symptomatology, anxiety symptoms, probable PTSD and resilience*.*

#### Sociodemographic characteristics

The demographic information of the participants included age, gender (male, female or other), school grade (for the Colombian education system, grades 6th to 9th correspond to basic secondary education, and grades 10th and 11th correspond to middle education, which ends with a bachelor's degree), municipality of residence (Chaparral, Ataco, Rioblanco or Planadas), number of years living in the municipality, people they live with (mother, father, siblings, grandparents, others) and number of people in the home.

#### Family functionality

Participants' family functioning was evaluated using the Family APGAR validated in Colombia which evaluate dimensions like adaptability, cooperation, development, affectivity, resolution capacity and commitment^22,23^. Examples of items include “I am satisfied with the help I receive from my family when I have a problem or need” and “I am satisfied with how my family expresses affection and responds to my emotions such as anger, sadness, love”. The scale is scored on a 5-point scale and consists of seven items, each of which is scored from 0 (never) to 5 (always). The total score ranges from 0 to 35. A score greater than 18 indicates normal family functioning, a score of 17–4 indicates mild family dysfunction, 13–10 moderate dysfunction, and less than 9 severe dysfunction.

#### Mental health symptoms

Mental health symptoms were reported by the participants through the Self-Report Questionnaire (SRQ) designed by the World Health Organisation (WHO) which allows screening for possible mental health problems^24^. SQR results are analysed based on the self-reported presence or absence of mental health symptoms categorised as positive or negative answers into four categories: the first block of 20 questions assesses anxious or depressive symptomatology, eleven or more positive answers suggest a high probability of mental health disorder whether it was depression, anxiety or both; the second block has 4 questions regarding psychotic symptomatology, followed by a third block with one question about epilepsy; and finally, the last 5 questions inquire about alcohol consumption where one or more positive answers suggest a high probability of problematic alcohol consumption. We focus on affective symptoms and alcohol consumption since, according to previous studies, the questions regarding psychotic symptoms can be misinterpreted in regions with armed conflict^17^.

#### Depressive symptomatology

Depressive symptomatology was assessed using the Patient Health Questionnaire (PHQ-8)^25^. It consists of eight items. The response options for each item range from not at all (0 points) to almost every day (3 points), reflecting how often each symptom has affected respondents in the past 2 weeks. As validated in the Colombian population, scores of 5, 10, 15 and 20 represent respectively cut-off points for mild, moderate, moderately severe and severe depressive symptomatology. A cut-off score of 10 or more based on the PHQ-8 is considered positive screening for depression. Those with a positive result should be evaluated clinically to determine a diagnosis of depressive disorder^15,16^.

#### Symptoms of anxiety

Anxiety symptoms were measured using the Colombian version of the Generalized Anxiety Disorder Scale (GAD-7)^26^, which consists of 7 items rated on a scale from 0 (any day) to 3 (almost every day), reflecting the frequency with which each symptom has been experienced by respondents in the last 2 weeks. As validated in the Colombian population, scores of 5, 10 and 15 represented cut-off points for mild, moderate and severe symptoms of anxiety, respectively. Positive screening for anxiety is a cut-off score of 10 or more based on GAD-7 and due is a screening tool, further clinical evaluation is recommended^17^.

#### Post-Traumatic Stress Disorder (PTSD)

Considering the historic violent context of Colombia in general and of the included municipalities in particular, the Post-Traumatic Stress Disorder Checklist for DSM-5 (PCL-5) was used to measure symptoms of post-traumatic stress disorder in those reported having experienced a traumatic event during the life^27^. It is a 20-item self-report measure that assesses the 20 DSM-5 PTSD symptoms in the last 30 days. Each item is rated on a scale from 0 (not at all) to 4 (extremely) reflecting how much the symptoms have bothered the respondent. PCL-5 provides a provisional diagnosis of PTSD. If the total of the global scale reaches a cut-off score of 33 or more, it is indicative of probable PTSD^22^.

#### Resilience

The Connor and Davidson Resilience Scale (CD-RISC-25) is designed to explore five factors related to resilience as personal competence, tolerance and strength, positiveness, control and spiritual influences^28^. Scoring of the scale is based on summing the total of all 25-items, each of which is scored from 0 (absolutely) to 4 (almost always), so the full range is from 0 to 100. Higher scores reflect greater resilience. The results are presented calculating the medians and interquartile ranges of each sample, which makes it possible to compare the resilience capacity between different populations^28^.

### Statistical analysis

Descriptive statistics were used for the total sample of the study, with subgroup analyses by sex, age, municipality and family functionality. Continuous variables are presented as mean (SD) or median [IQR] depending on whether the distribution is normal or not, and categorical data are reported as absolute and relative frequencies (percentages). Based on the recommended cut-offs, prevalence and 95% confidence intervals for depressive symptomatology, anxiety symptoms and probable PTSD were estimated with continuity correction. For the CD-RISC-25, the median and the observed distribution of the scores by quartile were determined. Statistical significance tests were performed for all conditions. Parametric or non-parametric tests were performed to assess the correlation between mental health conditions and the resilience scale, sociodemographic characteristics and family functionality. For the analysis of the results in the GAD-7, PHQ-8 and PCL-5 scales, the Pearson's chi-square test was performed and for the CD-RISC-25 the Kruskal Wallis test was performed, except for the comparison with the PCL-5 variable, where the U Mann Whitney Wilcoxon test was performed. Analyses were performed using the R software^®^ version 4.2.0.

### Ethical approval and consent to participate

The study was carried out in accordance with the ethical principles that govern research in Colombia and the United Kingdom (UK). The study protocol was evaluated and approved by Pontificia Universidad Javeriana Faculty of Medicine Institutional Research and Ethics Committee (CIEI-0730-20) and the Queen Mary University of London Research Ethics Committee (QMERC20.226). Written informed assent and consent was obtained by all the participants and their parents or legal guardians.

## Results

### Sociodemographic characteristics

Table 1 displays the sociodemographic characteristics of the 657 school-attending adolescents included in this study. The average age of participants was 14 years (SD 1.9), 57.4% identified themselves as female and 41.9% identified as male. Students in basic secondary education represented 76.7% of participants, and those in middle education represented 23.3% of our total sample. Most adolescents were in the 8th and 6th grade (26.9% and 18%, respectively). The distribution of the sample by municipalities corresponds to the number of inhabitants in each of them. Chaparral was the municipality with the largest sample (54.5%) and Ataco the municipality with the smallest sample (14.2%). The average of time living in the current municipality was 10 years.

**Table 1:** Sociodemographic characteristics of participants.

Characteristic	n (%)
Mean age (SD)	14.0 (1.9)
Gender	
Male	275 (41.9)
Female	377 (57.4)
Other	5 (0.8)
School grade	
6th grade	118 (18.0)
7th grade	102 (15.5)
8th grade	177 (26.9)
9th grade	107 (16.3)
10th grade	80 (12.2)
11th grade	73 (11.1)
Municipality of residence	
Chaparral	358 (54.5)
Ataco	93 (14.2)
Rioblanco	108 (16.4)
Planadas	98 (14.9)
Years of residence-mean (SD)	10.0 (4.9)

### Family functionality

Regarding family structure, 40.8% of participants reported living with their mothers, 6.2% with their fathers, 42.8% with both parents and 10.2% with neither of them. The mean number of people living in their households was four. Family functionality was evaluated with the family APGAR. Table 2 shows these results according to age, gender and municipality. It was found that 35.2% of participants had Family APGAR scores suggesting some type of family dysfunction and, of these, one in four reported mild and moderate dysfunction. Among participants who reported severe family dysfunction, 30.2% were male whilst 62.8% were female. Participants between 13 and 16 years of age had higher prevalence of some degree of family dysfunction. Chaparral and Planadas were the municipalities where severe family dysfunction was most likely to be reported.

**Table 2:** Family APGAR.

Characteristics	Family APGAR (n (%))			
	No dysfunction (n = 426)	Mild dysfunction (n = 118)	Moderate dysfunction (n = 70)	Severe dysfunction (n = 43)
Age				
≤ 12 years old	121 (28.4)	21 (17.8)	10 (14.3)	8 (18.6)
13–14 years old	154 (36.2)	46 (39.0)	27 (38.6)	15 (34.9)
15–16 years old	115 (27.0)	32 (27.1)	21 (30.0)	14 (32.6)
≥ 17 years old	36 (8.5)	19 (16.1)	12 (17.1)	6 (14.0)
Gender				
Male	189 (44.4)	44 (37.3)	29 (41.4)	13 (30.2)
Female	236 (55.4)	73 (61.9)	41 (58.6)	27 (62.8)
Other	1 (0.2)	1 (0.8)	0 (0.0)	3 (7.0)
Municipality				
Chaparral	234 (54.9)	61 (51.7)	36 (51.4)	27 (62.8)
Ataco	60 (14.1)	16 (13.6)	13 (18.6)	4 (9.3)
Rioblanco	74 (17.4)	21 (17.8)	10 (14.3)	3 (7.0)
Planadas	58 (13.6)	20 (16.9)	11 (15.7)	9 (20.9)

### Mental health symptoms

These results were obtained with the Self-Reporting Questionnaire (SRQ) and showed that 23.9% of participants had 11 or more positive answers in anxious and/or depressive symptomatology. The highest percentage of positive answers was obtained for the 15th item of the questionnaire, where 60.9% of participants declared having lost interest in things, followed by 51.8% of adolescents who answered ‘yes’ to having difficulty making decisions. A similar proportion of adolescents (49.0%) reported ‘yes’ to feeling nervous, tense or worried. Of great concern, items assessing alcohol consumption, showed that 113 participants, representing 17.2% of our total sample, were at high-risk alcohol consumption.

### Depressive symptomatology

As seen in Table 3, scores on the Patient Health Questionnaire depression scale (PHQ-8) indicated some degree of depressive symptomatology in 59.8% of participants. The prevalence for moderate to severe depressive symptomatology was 30% (95% CI 26.5–33.7), increasing to 59.8% (95% CI 55.9–63.6) when mild depressive symptomatology was included.

**Table 3:** PHQ-8 sociodemographic characteristics and family functionality.

PHQ-8—depressive symptomatology (n (%))						p value
Characteristics	None 264 (40.2)	Mild 196 (29.8)	Moderate 108 (16.4)	Moderately severe 60 (9.1)	Severe 29 (4.4)	
Age						
≤ 12 years old	82 (51.2)	45 (28.1)	22 (13.8)	6 (3.8)	5 (3.1)	0.055
13–14 years old	95 (39.3)	71 (29.3)	42 (17.4)	24 (9.9)	10 (4.1)	
15–16 years old	64 (35.2)	55 (30.2)	35 (19.2)	19 (10.4)	9 (4.9)	
≥ 17 years old	23 (31.5)	25 (34.2)	9 (12.3)	11 (15.1)	5 (6.8)	
Gender						
Male	141 (51.3)	76 (27.6)	38 (13.8)	14 (5.1)	6 (2.2)	< 0.001
Female	122 (32.4)	120 (31.8)	70 (18.6)	43 (11.4)	22 (5.8)	
Other	1 (20.0)	0 (0.0)	0 (0.0)	3 (60.0)	1 (20.0)	
School grade						
6th	61 (51.7)	32 (27.1)	14 (11.9)	6 (5.1)	5 (4.2)	0.279
7th	42 (41.2)	32 (31.4)	20 (19.6)	6 (5.9)	2 (2.0)	
8th	71 (40.1)	54 (30.5)	29 (16.4)	14 (7.9)	9 (5.1)	
9th	40 (37.4)	30 (28.0)	15 (14.0)	15 (14.0)	7 (6.5)	
10th	29 (36.2)	22 (27.5)	15 (18.8)	10 (12.5)	4 (5.0)	
11th	21 (28.8)	26 (35.6)	15 (20.5)	9 (12.3)	2 (2.7)	
Municipality						
Chaparral	134 (37.4)	108 (30.2)	66 (18.4)	35 (9.8)	15 (4.2)	0.240
Ataco	50 (53.8)	27 (29.0)	8 (8.6)	5 (5.4)	3 (3.2)	
Rioblanco	48 (44.4)	28 (25.9)	17 (15.7)	10 (9.3)	5 (4.6)	
Planadas	32 (32.7)	33 (33.7)	17 (17.3)	10 (10.2)	6 (6.1)	
Family APGAR						
Normal	214 (50.2)	131 (30.8)	49 (11.5)	24 (5.6)	8 (1.9)	< 0.001
Mild	31 (26.3)	38 (32.2)	29 (24.6)	12 (10.2)	8 (6.8)	
Moderate	11 (15.7)	17 (24.3)	17 (24.3)	16 (22.9)	9 (12.9)	
Severe	8 (18.6)	10 (23.3)	13 (30.2)	8 (18.6)	4 (9.3)	

Regarding gender distribution, moderate and severe depressive symptomatology was reported by 35.8% of females and by 21.8% of males. 51.3% males versus 32.4% females reported no depressive symptomatology. As the level of schooling increased, the proportion of adolescents with no depressive symptomatology decreased. The sixth graders, had the lowest proportion for mild, moderate and moderately severe depressive symptomatology. Meanwhile, the highest proportion of mild and moderate depressive symptomatology was seen for the eleventh-grade adolescents, with 35.6% and 20.5%, respectively. The relative frequency for moderately severe (14.0%) and severe (6.5%) depressive symptomatology was higher for the 9th grade students.

Geographically, Planadas was the municipality where participants had the highest proportion of depressive symptomatology, with 33.7% of adolescents reporting mild symptoms. Similarly, moderate and severe depressive symptomatology were reported by 33.6% in this region. This was followed by the municipality of Chaparral, where 32.4% of adolescents reported moderate and severe depressive symptomatology. The lowest proportion of depressive symptomatology was reported in Ataco, where mild symptoms had higher percentages than moderate and severe forms.

Severe depressive symptomatology was present in 44.8% of participants who reported moderate or severe family dysfunction. Of participants with some degree of depressive symptomatology, only 47.8% reported adequate family functionality.

### Symptoms of anxiety

Results from the Generalized Anxiety Disorder 7-item (GAD-7) scale, as seen in Table 4, showed that 29.5% of adolescents reported mild anxiety symptoms, whilst moderate and severe symptomatology was reported by 11.7% and 7.2% of participants, respectively. The prevalence observed for moderate to severe symptoms of anxiety was 18.9% (95% CI 16.0–22.1). Including mild symptoms increased the prevalence for anxiety to 48.4% (95% CI 44.5–52.3).

**Table 4:** GAD-7 sociodemographic characteristics and family functionality.

GAD-7—anxiety [n (%)]					p value
Characteristic	None	Mild	Moderate	Severe	
	339 (51.6)	194 (29.5)	77 (11.7)	47 (7.2)	
Age					
≤ 12 years old	101 (63.1)	34 (21.2)	18 (11.2)	7 (4.4)	0.019
13–14 years old	127 (52.5)	73 (30.2)	26 (10.7)	16 (6.6)	
15–16 years old	82 (45.1)	58 (31.9)	26 (14.3)	16 (8.8)	
≥ 17 years old	29 (39.7)	29 (39.7)	7 (9.6)	8 (11.0)	
Gender					
Male	179 (65.1)	62 (22.5)	24 (8.7)	10 (3.6)	< 0.001
Female	160 (42.4)	129 (34.2)	52 (13.8)	36 (9.5)	
Other	0 (0.0)	3 (60.0)	1 (20.0)	1 (20.0)	
School grade					
6th	74 (62.7)	26 (22.0)	11 (9.3)	7 (5.9)	0.035
7th	65 (63.7)	19 (18.6)	12 (11.8)	6 (5.9)	
8th	86 (48.6)	59 (33.3)	18 (10.2)	14 (7.9)	
9th	52 (48.6)	30 (28.0)	16 (15.0)	9 (8.4)	
10th	34 (42.5)	31 (38.8)	11 (13.8)	4 (5.0)	
11th	28 (38.4)	29 (39.7)	9 (12.3)	7 (9.6)	
Municipality					
Chaparral	182 (50.8)	110 (30.7)	36 (10.1)	30 (8.4)	0.149
Ataco	55 (59.1)	27 (29.0)	7 (7.5)	4 (4.3)	
Rioblanco	57 (52.8)	24 (22.2)	19 (17.6)	8 (7.4)	
Planadas	45 (45.9)	33 (33.7)	15 (15.3)	5 (5.1)	
Family APGAR					
Normal	262 (61.5)	118 (27.7)	32 (7.5)	14 (3.3)	< 0.001
Mild	47 (39.8)	37 (31.4)	19 (16.1)	15 (12.7)	
Moderate	20 (28.6)	25 (35.7)	12 (17.1)	13 (18.6)	
Severe	10 (23.3)	14 (32.6)	14 (32.6)	5 (11.6)	

Age distribution of anxiety symptoms showed that older adolescents (17 years of age) had a higher proportion of mild (39.7%) and severe (11.0%) symptoms, whilst the relative frequency of moderate symptoms was higher in adolescents between 15 and 16 years old. A larger proportion of females reported anxiety symptoms. Considering school grades, adolescents enrolled in the 9th grade had a higher proportion of moderate symptoms and, matching the results observed by age distribution, those at the 11th grade had higher relative frequencies in the mild and severe categories. Regarding the different municipalities, the proportion of adolescents with mild symptomatology was highest in Planadas (33.7%). Rioblanco reported higher relative frequency in the moderate category (17.6%). Adolescents living in Chaparral had the highest proportion of adolescents in the severe category (8.4%). Severe anxiety symptoms were found in 38.3% of adolescents with moderate and severe family dysfunction.

### Post-Traumatic Stress Disorder (PTSD)

The Post-Traumatic Stress Disorder Checklist for DSM-5 (PCL-5)^29^ was used in order to determine the psychological effects of traumatic experiences. 51.2% of participants reported having experienced at least one traumatic event during their lifetime. The most frequent reported traumatic events were related to accidents, physical, psychological and sexual violence, common crime and armed conflict.

The results in PCL-5 showed a prevalence of probable Post-Traumatic Stress Disorder (PTSD) of 22.3% (95% CI 18.1–27.2), using the cut off of 33 or more. As show in Table 5, regarding age distribution, 70.6% of adolescents between 13 and 16 years of age reported possible symptoms of PTSD. As seen with the anxious and depressive symptomatology, a higher proportion of females (26.2%) reported probable PTSD symptoms versus males (15.3%). Regarding municipalities, 24.8% and 20.8% of adolescents with probable diagnosis of PTSD lived in Chaparral and Rioblanco, respectively. Regarding family functionality, 53.3% adolescents with a probable PTSD diagnosis reported severe family dysfunction.

**Table 5:** PCL-5, sociodemographic characteristics and family functionality.

	PCL-5-probable PTSD (n (%))	p value
Age		
≤ 12 years old	13 (17.6)	0.620
13–14 years old	29 (23.6)	
15–16 years old	24 (25.5)	
≥ 17 years old	9 (20.0)	
Gender		
Male	21 (15.3)	0.018
Female	51 (26.2)	
Other	3 (75.0)	
School grade		
6th	15 (24.6)	0.612
7th	9 (18.0)	
8th	18 (22.2)	
9th	16 (28.6)	
10th	7 (14.9)	
11th	10 (24.4)	
Municipality		
Chaparral	51 (24.8)	0.521
Ataco	5 (14.7)	
Rioblanco	10 (20.8)	
Planadas	9 (18.8)	
Family APGAR		
Normal	30 (15.2)	< 0.001
Mild	17 (25.8)	
Moderate	12 (27.9)	
Severe	16 (53.3)	

### Symptoms of depression, anxiety and PTSD

Looking at the results as a whole, 66.2% of adolescents had at least one mental health problem like depressive symptomatology, symptoms of anxiety or probable PTSD. The proportion of adolescents with positive screening for anxious and depressive symptomatology was 32.7%, whilst 9.9% of participants had positive symptoms for anxiety and probable PTSD. Only 33.8% of adolescents surveyed reported no symptoms of depression, anxiety or PTSD.

### Resilience

For the CD-RISC-25 in our sample (n = 657), the median was 54 [39–69] and the observed distribution of the scores by quartile was Q1 [0–39], Q2 [40–54], Q3 [55–69] and Q4 [70–100].

As seen in Table 6, the relationship between CD-RISC-25 scores and gender was not significant. The median was 54 [5–39; 39–63] for males and 55 [39–68] for females (p = 0.994). In relation to age, we found lower CD-RISC-25 scores in adolescents under 12 years of age compared to those between 15 and 16 years of age. These medians were 50 [33; 65] and 58.5 [43; 72.75] (p = 0.001), respectively. Students in the sixth grade (51.5 [34; 65.75]) had significantly lower scores than those in the eleventh grade (61 [51;74]) (p = 0.007). Those with moderate or severe family dysfunction reported lower resilience scores.

**Table 6:** Resilience scale CD-RISC.

	Resilience scale CD-RISC Median [Q1; Q3]	p value
GAD-7		
None	54 [35.5;69]	0.221
Mild	56 [44; 70]	
Moderate	52 [39; 61]	
Severe	48 [38; 61]	
PHQ-8		
None	56 [36; 72]	0.007
Mild	55 [42.75; 69]	
Moderate	55 [40; 68.25]	
Moderately severe	47 [37; 58]	
Severe	46 [35; 56]	
PCL-5		
None	55 [41; 70]	0.136
Possible PTSD	54 [37; 63]	
Age		
≤ 12 years old	50 [33;65]	0.001
13–14 years old	52 [40; 66]	
15–16 years old	58.5 [43; 72.75]	
≥ 17 years old	57 [44; 71]	
Gender		
Male	54 [39.5; 69]	0.994
Female	55 [39; 68]	
Other	49 [46; 56]	
School grade		
6th	51.5 [34; 65.75]	0.007
7th	51 [36; 64]	
8th	53 [40, 68]	
9th	54 [40.5; 67.5]	
10th	59 [40.75; 74.25]	
11th	61 [51; 74]	
Family APGAR		
Normal	57 [42.25; 71]	< 0.001
Mild	54 [39.25; 65]	
Moderate	46.5 [34; 58.75]	
Severe	44 [27.5; 54]	

Regarding mental health problems, those with the most severe forms of depressive and anxious symptomatology reported lower resilience scores. The percentage of those reporting a probable diagnosis of PTSD on the PCL-5 was similar to those who did not present this possible diagnosis.

## Discussion

The vast effects that the COVID-19 pandemic had on the mental health of the adolescent population worldwide have to be carefully evaluated, and multidisciplinary efforts are required in order to address the psychological, educational and social problems that have arisen^29^. These efforts are particularly important in settings where poverty, armed conflict and internal displacement already placed a burden on the mental wellbeing of the population^30–32^.

Although the long-term impact of school closings, social distancing and other lockdown measures are emerging, it has been acknowledged that losses in learning and delay in cognitive development may be intensifying inequalities and halting future life possibilities for today’s younger generation^5,15,30^. In the case of Colombia, this is especially important in rural areas where the high impact of the armed conflict has led to state abandonment, limiting the population's access to education, health, social services and technological resources^19,33^. In this scenario, ensuring continuity in education during the pandemic was a challenge both for schools and for students and their families.

National statistics show that, between 2020 and 2021, school dropout and grade repetition rates increased, and gaps in academic achievement increased^34^. In our study, we found a low number of students, both female and male, in middle education, compared to those in basic education. Although this is in line with other findings where vulnerable populations have less schooling at higher grades—often due to the need to work or to carry out other income-generating activities, teenage pregnancies, or having to care for a family member—it is likely that the economic impact of the pandemic and the restructuring of families due to deaths related to COVID-19 have increased these needs and may have forced adolescents into abandoning their education^4,35,36^. Additional research in this regard would allow us to learn more about the psychosocial impact of the pandemic on adolescent schooling.

The emerging evidence globally suggests that multiple domains of adolescent psychosocial wellbeing have been negatively affected by the pandemic, while studies from other regions affected by armed conflict have found higher prevalence of mental health disorders in young populations^5,7,30,37,38^.

This study showed that approximately two-thirds of school-attending adolescents living in post-conflict area in Colombia experienced during the COVID-19 pandemic at least one mental health problem such as anxiety symptoms, depressive symptomatology or probable PTSD.

In Colombia, according to the NMHS-2015, the lifetime prevalence of any mental health disorder in adolescents between 12 and 17 years of age was of 7.2%, and positive affective symptoms on the SRQ were found in 12.2% of adolescents^17,39^. Our findings show an almost two-fold increase, where 23.9% of participants had positive answers in symptomatology associated with depressive and anxiety in the SRQ.

Interestingly, in analysing individual SRQ questions, our results showed that the vast majority of adolescents answered positively to having lost interest in things. With this in mind, a previous study in Latin America reported that half of adolescents felt less motivated to do activities that they previously enjoyed and have also reported a pessimistic perception of the future, which suggests that feelings of hopelessness might be more common after the pandemic in this population^40^.

Regarding depression and anxiety disorders measured by the NMHS-2015, there found a higher prevalence of anxiety disorders, with an estimated lifetime prevalence of 5% compared to 2.4% of any depressive disorder. Conversely, using standard cut-offs, our sample showed a remarkably higher prevalence of positive screening of depressive symptomatology (30.0%) according to the PHQ-8, compared to 18.9% prevalence observed using the GAD-7 to screen for anxiety symptoms.

Worldwide, the prevalence of depression and anxiety disorders have a broad range, but international studies seem to agree that, compared to pre-pandemic estimates, the mental health problems of adolescents have likely doubled. A 2021 meta-analysis revealed that around 1 in 4 adolescents globally might be experiencing depression symptoms and 1 in 5 are facing clinically elevated symptoms of anxiety^37^. Additionally, it is important to note that emerging studies, along with ours, show that older adolescents have higher depressive and anxious symptomatology, throughout female adolescents show a higher prevalence^5,37,41^.

A recent study performed by Jones and colleagues in vulnerable adolescents living in Jordan (including Syrian refugees, Palestinians and Jordanians) using mixed-methods analysis found that 19.3% of adolescents presented moderate to severe depressive symptomatology^7^. This is lower than our results where 30.0% of adolescents reported these levels of symptoms through a screening scale, perhaps after clinical assessment the prevalence may be similar. However, our findings correlate with those found in the adolescent population in areas of armed conflict in Colombia where depressive symptomatology is increased by violence and relational difficulties, which were probably aggravated during the pandemic^42^. Interestingly, the follow-up for Jones and colleagues’ study showed that school-attending adolescents had greater decreases in depressive symptoms compared to out of school adolescents. Another study geographically diverse, which include Perú, a country culturally and socioeconomically closer to our context, revealed higher rates of depression and anxiety compared to Ethiopia, India and Vietnam. Regarding mild depressive symptomatology, similar prevalence was found: 32% for Perú compared to 29.8% in our Colombian study. However, moderate and severe depressive symptomatology in our study was almost three times as high, with 30.0% versus 9.6% in Perú^41^. These differences may be related to the moment of the pandemic in which the sample was collected, in the Peruvian study the sample was taken in the first months of the pandemic and ours almost 2 years later, where the mild symptoms could have worsened.

Anxiety symptoms for the adolescent population in Jordan showed moderate to severe symptoms of anxiety in 12.4% of adolescents. This finding, as well as their depressive symptomatology estimates, is lower than our finding of 18.9% for the same categories. On the other hand, the estimated prevalence of symptoms of mild anxiety in Peruvian adolescents was of 41%, higher than our estimates of 29.5%. They calculated a 13.5% prevalence for symptoms of moderate and severe anxiety, while our results showed higher estimates of 18.9%^42^. Probably, contrary to depressive symptomatology, mild anxiety symptoms may have improved as the pandemic progressed.

Previous studies have shown that adolescents with a prolonged exposure to armed conflict and to the economic and sociocultural conditions typical of these contexts have a high risk of mental health issues. It has been found that, in these populations, violence and depression are the first and third leading cause of disability-adjusted life years^43,44^. Regarding traumatic events, 51.2% of all adolescents in our sample reported having experienced at least one traumatic event during their lifetime compared to the NMHS-2015 result where this was only 29.3%^19^. It is known that the risk of PTSD increases in times of pandemics as well as in disasters^45^. In Colombia, although the NMHS-2015 had no precise estimate of PTSD risk in adolescents, the prevalence of individual symptoms was calculated between 6.6 and 19.4% using the PCL-C. Our study using the PCL-5 and focus in a region with high impact of armed conflict shows higher estimates, with a prevalence of probable diagnosis of PTSD of 22.32%. Internationally, findings are variable regarding PTSD and the COVID-19 outbreak in adolescents, since most studies focus on adult populations. Different studies performed in Turkey and Saudi Arabia found prevalence of potential PTSD in 28.5% and 13% of adolescents, respectively. A study conducted in one of the least COVID-affected provinces of China, found a PTSD prevalence of 3.1% in 8–13 year old children^46–48^. These differences may be related to the particular context of the country, exposure to family and socioeconomic stressors and the resilience of populations.

Another factor which heavily influences the mental health of adolescents is family functionality, and low family functioning is strongly related to poor adolescent mental health^49^. Adolescents in our sample who reported depressive and anxious symptomatology, also showed varying degrees of family dysfunction. The family environment has been particularly affected during the pandemic, with global studies reporting increased rates of domestic violence and strained parent–child relationships^50–52^. This also affects the likelihood of substance abuse: a Swedish study showed that adolescents with higher family conflict and tense relationship with parents had higher risks of substance abuse^53^.

Our study also showed a remarkable percentage of adolescents with high-risk alcohol consumption (17.2%), as measured by the SRQ-30. Previous studies have shown that, for the adolescent population, alcohol consumption is the main risk factor for presenting disability-adjusted life years^44^. Our findings showed a more than three-fold increase compared with the results obtained in the NMHS-2015, where 5.2% of adolescents had an excessive alcohol consumption as measured with the AUDIT-C. A similar outcome was found in Indonesian adolescents where alcohol consumption during the pandemic had a two-fold increase compared to pre-pandemic rates^54^. In the United States, a study showed that the prevalence of alcohol consumption in adolescents did not change significantly, but the frequency of alcohol use increased among those who already drank alcohol^55^.

Regarding resilience, our results showed a median CD-RISC-25 of 54 [IQR 30]. This is similar to the mean result of 54.7 obtained in displaced Iraqi adolescents pre-pandemic^56,57^, and represents a low resilience level compared to studies performed in adolescent populations exposed to traumatic experiences in Australia and the USA, where CD-RISC mean results were 62.2 and 69.8, respectively^27,58,59^. As seen in most studies, older adolescents had higher resilience than their younger counterparts, and it is noteworthy that those with a probable PTSD diagnosis in our sample did not have low CD-RISC-25 scores. This could be related to observations from other studies where resilience is higher in populations that have been chronically exposed to violence and armed conflict, like adolescent Syrian refugees living in camps and Liberian adolescents^7,60,61^.

These findings, in addition to the evidence of new adversities imposed by the COVID-19 pandemic, constitute a call for urgent action to mitigate this problematic experience for adolescents who live in these contexts. It also highlights the importance that educational and school settings have in their mental health.

The worldwide closure of schools and learning institutions has no recent precedent^62^. Online and remote learning was used as an alternative in most countries, however, this option often excluded those who live in poverty, those in rural areas with limited infrastructure and especially those with no access to internet^62^. This could potentially exacerbate existing inequalities and in turn reduce the learning potential of a generation of adolescents^62^. Furthermore, emotional, behavioural and psychosocial problems that arise during childhood and adolescence cause major disruption in the learning process. This leads to low academic performance and school dropout, which then translates into negative repercussions for their futures. According to UNICEF, around 1.6 billion adolescents globally have experienced some educational loss since the beginning of the pandemic^63^.

Multidisciplinary efforts and actions are required to effectively respond to the impact of the COVID-19 pandemic on the mental health of adolescents, as well as to guarantee a successful educational recovery. Our findings highlight the great challenge that schools are facing now that the students returned to in-person classes to address the mental health of their students in order to promoting adequate coping strategies and implement prompt multidisciplinary interventions to reduce the burden of mental health problems in adolescents.

To the best of our knowledge, our study is the first to estimate the mental health challenges during the COVID-19 pandemic in the adolescent population of this particularly vulnerable setting that has been affected by the armed conflict. Our results are consistent with emerging studies that highlight the increased mental health struggle of vulnerable populations. Considering the study design, it is not possible to establish a causal relationship between the findings and the impact of the pandemic and armed conflict on adolescent's mental health. However, these findings highlight the importance of conducting future studies to characterise this relationship. Additionally, our sample is not nationally representative and included only school-attending adolescents. Therefore, the results should be interpreted with caution. Further research is still needed in order to fully grasp the effects of armed conflict and the pandemic on adolescents not attending school. This group that, due to different circumstances, had to stop their education, might face even more mental health problems than their school-attending peers. The point prevalence we report were measured once students returned to school after the closures imposed by the pandemic. Thus, these results could be influenced by the time of measurement. Further studies on longer-term effects on mental health and on future outcomes on adolescent development are urgently needed.

## Conclusions

This study showed that approximately two-thirds of school-attending adolescents living in post-conflict area in Colombia experienced during the COVID-19 pandemic at least one mental health problem such as anxiety symptoms, depressive symptomatology or probable PTSD. Severe depressive and anxious symptomatology was associated to lower resilience. Future studies are needed to establish the causal relationship between these results and the impact of armed conflict and the COVID-19 pandemic on adolescent mental health. These findings highlight the great challenge that schools are facing now to address the mental health of their students. This is a call to increase understanding of the importance of mental health in the school settings and to implement strategies that promote, protect, and restore the mental health of their students. Creating a positive mental health space in schools where mental health is openly discussed reduces stigma and encourages self-care habits, while also allowing students to share their challenges and ask for help. A curriculum that includes social and emotional learning, as well as life skills, helps to strengthen a sense of control, and improves resilience and coping skills. Additionally, training teachers, principals and school counsellors to play active roles to promote mental well-being, prevention, identification and prompt intervention in common mental health problems could further reduce the burden of mental health problems in adolescents.

## Data Availability

The datasets used and analysed during the current study are available from the corresponding author on reasonable request.

## Abbreviations

APGAR: Family adaptability, partnership, growth, affection and resolve measure
CD-RISC-25: 25-Item Connor Davidson Resilience Scale
FARC-EP: Spanish acronym for Revolutionary Armed Forces of Colombia-People’s Army
GAD-7: Generalized Anxiety Disorder 7-item anxiety scale
NMHS-2015: National Mental Health Survey, 2015, Colombia
PCL-5: Post-Traumatic Stress Disorder Checklist
PDET: Spanish acronym for Development Program with a Territorial Approach
PHQ-8: Patient Health Questionnaire depression scale
PTSD: Post-Traumatic Stress Disorder
SRQ: Self-Reporting Questionnaire
